# Fractional-Order SEIQRDP Model for Simulating the Dynamics of COVID-19 Epidemic

**DOI:** 10.1109/OJEMB.2020.3019758

**Published:** 2020-08-26

**Authors:** Mohamed A. Bahloul, Abderrazak Chahid, Taous-Meriem Laleg-Kirati

**Affiliations:** Computer, Electrical, and Mathematical Sciences, and Engineering Division (CEMSE)King Abdullah University of Science, and Technology (KAUST) 127355; Computer, Electrical, and Mathematical Sciences, and Engineering Division (CEMSE)King Abdullah University of Science, and Technology (KAUST) 127355 Thuwal 23955-6900 Makkah Province Saudi Arabia

**Keywords:** Coronavirus, COVID-19, fractional-order derivative, pandemic, SEIR models

## Abstract

*Goal:* Coronavirus disease (COVID-19) is a contagious disease caused by a newly discovered coronavirus, initially identified in the mainland of China, late December 2019. COVID-19 has been confirmed as a higher infectious disease that can spread quickly in a community population depending on the number of susceptible and infected cases and also depending on their movement in the community. Since January 2020, COVID-19 has reached out to many countries worldwide, and the number of daily cases remains to increase rapidly. *Method:* Several mathematical and statistical models have been developed to understand, track, and forecast the trend of the virus spread. *Susceptible-Exposed-Infected-Quarantined-Recovered-Death-Insusceptible (SEIQRDP)* model is one of the most promising epidemiological models that has been suggested for estimating the transmissibility of the COVID-19. In the present study, we propose a fractional-order SEIQRDP model to analyze the COVID-19 pandemic. In the recent decade, it has proven that many aspects in many domains can be described very successfully using fractional order differential equations. Accordingly, the Fractional-order paradigm offers a flexible, appropriate, and reliable framework for pandemic growth characterization. In fact, due to its non-locality properties, a fractional-order operator takes into consideration the variables’ memory effect, and hence, it takes into account the sub-diffusion process of confirmed and recovered cases. *Results–*The validation of the studied fractional-order model using real COVID-19 data for different regions in China, Italy, and France show the potential of the proposed paradigm in predicting and understanding the pandemic dynamic. *Conclusions:* Fractional-order epidemiological models might play an important role in understanding and predicting the spread of the COVID-19, also providing relevant guidelines for controlling the pandemic.

## I. Introduction

Covid-19 is an illness caused by the new coronavirus that was first identified in Wuhan, Hubei province, China, late December 2019 [1]. The novel virus soon began to spread out around the world, and on 30 January, the World Health Organization (WHO) declared the outbreak as a public health emergency of international concern (PHEIC). On 11 March, WHO director-general marked COVID-19 as a pandemic [2]–[4]. COVID-19 is a higher infectious disease that can readily spread in a community population depending on the number of susceptible and infected cases and also depending on their movement in the community. The transmission of COVID-19 is primarily through respiratory droplets and contact routes [5]. It is primarily spread from person to person. A person becomes infected by coming into close contact (about 6 feet or two arm lengths) with a person who has COVID-19. He may also be able to get the virus by touching a surface or object that has the virus on it and then by touching his mouth, nose, or eyes. Currently, there is no vaccine to protect against COVID-19. The best way to protect ourselves is to avoid being exposed to the virus. Accordingly, ideal interventions to control the spread include: Clean and disinfect frequently touched surfaces, wash hands often for at least 20 seconds, quarantine, isolation, increase home confinement, promoting the wearing of face masks, travel restrictions, the closing of public space, and cancellation of events. Generally, everyone is at risk of getting COVID-19, but older adults and people of any age who have severe underlying medical conditions may be at higher risk for more severe illness. The number of cases increased rapidly to more than 3.25 million cases, including around 231,000 deaths worldwide as of April, 30, 2020.

With the surge of coronavirus disease contamination grows, it is vital to understand its dynamic as much as possible and estimate its key transmission parameters. Accordingly, since the outbreak began, COVID-19 has attracted the attention of scientists from different backgrounds, ranging from epidemiology to data science, mathematics, and statistics. Numerous investigations based on either statistics or mathematical modeling approaches have been proposed for better analysis and a deep understanding of the evolution of this pandemic. Besides, enormous efforts have been devoted to predicting the inflection point and ending time of this pandemic to help make decisions concerning the different measures taken by governments. In epidemiology, mathematical modeling has played a pivotal role in understanding and predicting the spread of the virus, also providing relevant guidelines for controlling the pandemic, [6]–[9]. In this regard, analytical models are considered to be very important as they provide reliable information on the pandemic dynamic and its effect on the community. Amongst the most widely studied model for the characterization of the COVID-19 outbreak in the world is the classic *Susceptible-Exposed-Infectious-Recovered* (SEIR) model [1], [10]. Since the outbreak of the virus, the SEIR model has been intensively utilized to evaluate the effectiveness of multiple measures, which seems to be a challenging task for general other estimation methods [11]–[13]. It has been employed to assess the effects of lock-down on the transmission dynamics of the virus between provinces in China, such as the effect of the lock-down in Hubei province on the transmission in Wuhan and Beijing [13]. In addition, a cascading scheme of the SEIR model has been investigated to emulate the manner of transmission from infection sources to humans. This approach was efficient to reach useful conclusions on the outbreak dynamics [14]. The work of [15] presents a generalization of the classical SEIR known as *Susceptible-Exposed-Infected-Quarantined-Recovered-Death-Insusceptible* (SEIQRDP) model, for the epidemic analysis of COVID-19 in China. In this generalization, new subpopulations have been added: protected (P), quarantine (Q), and dead (D). This generalization supposes that the susceptible population (S) can decrease due to the impact of restrictive government policies, such as lock-down and the mandatory wearing of face masks in public. Accordingly, apart of the susceptible category ($\alpha \cdot
                    S$) might be considered as a protected sub-population (P), where $\alpha$ represents the protection rate [16]. Besides, this model adds another category representing quarantined individuals (Q). Normally, following the infected persons have been tested positive, they will be kept out from the infective sub-population (I) and placed in quarantine. Indeed, they are supposed to be isolated and not to have had any contact with others. In addition, the generalized model separates the sub-populations of recovered (R) and dead (D) individuals. Generally, SEIQRDP considers the effect of preventive actions, which are considered crucial epidemic parameters for COVID-19, such as the latent and quarantine time. As a result, by utilizing this generalization of SEIR, the estimation of the inflection point, ending time, and total infected cases in extensively affected regions were determined and verified accurately. Although, SEIR models are potential paradigms in the dynamic epidemiological analyses and characterization, most of are restricted to integer-order (delay) differential equations. Recently, the fractional-order derivative (FD), defined as a generalization of the conventional integer derivative to a non-integer order (arbitrary order) operator, has been adopted to simulate many phenomena involving memory and delays including epidemic behavior [17]–[21]. FD models offer a promising tool for the description of complex systems. In addition to their potential to accurately incorporate the memory and delay involved in the systems, it also provides more flexibility than classical integer-order models in fitting the data [22].

In this paper, we present a fractional-order (*SEIQRDP*) modeling the COVID-19 pandemic spread in different regions in China, Italy, and France. We use real data to fit the model and examine its performance comparing to the integer-order SEIQRDP [20].

The rest of the paper is organized as follows. In Section II, we recall some basic concepts from the $SEIQRDP$ epidemic model and the fractional-order derivatives. In addition, it represents the proposed fractional-order (*SEIQRDP*) epidemic model (*F-SEIQRDP*). Section III exhibits the estimation results. The last section discusses the obtained results and limitation provides some future directions on the use of the model for analyzing and controlling the COVID-19 epidemic.

## II. Materials And Methods

### A. Preliminaries

In this part, we recall some basic concepts of the *SEIQRDP* epidemic model and the fractional-order derivatives theory.

#### 1) Generalized Epidemic Model: SEIQRDP

**Fig. 1.**
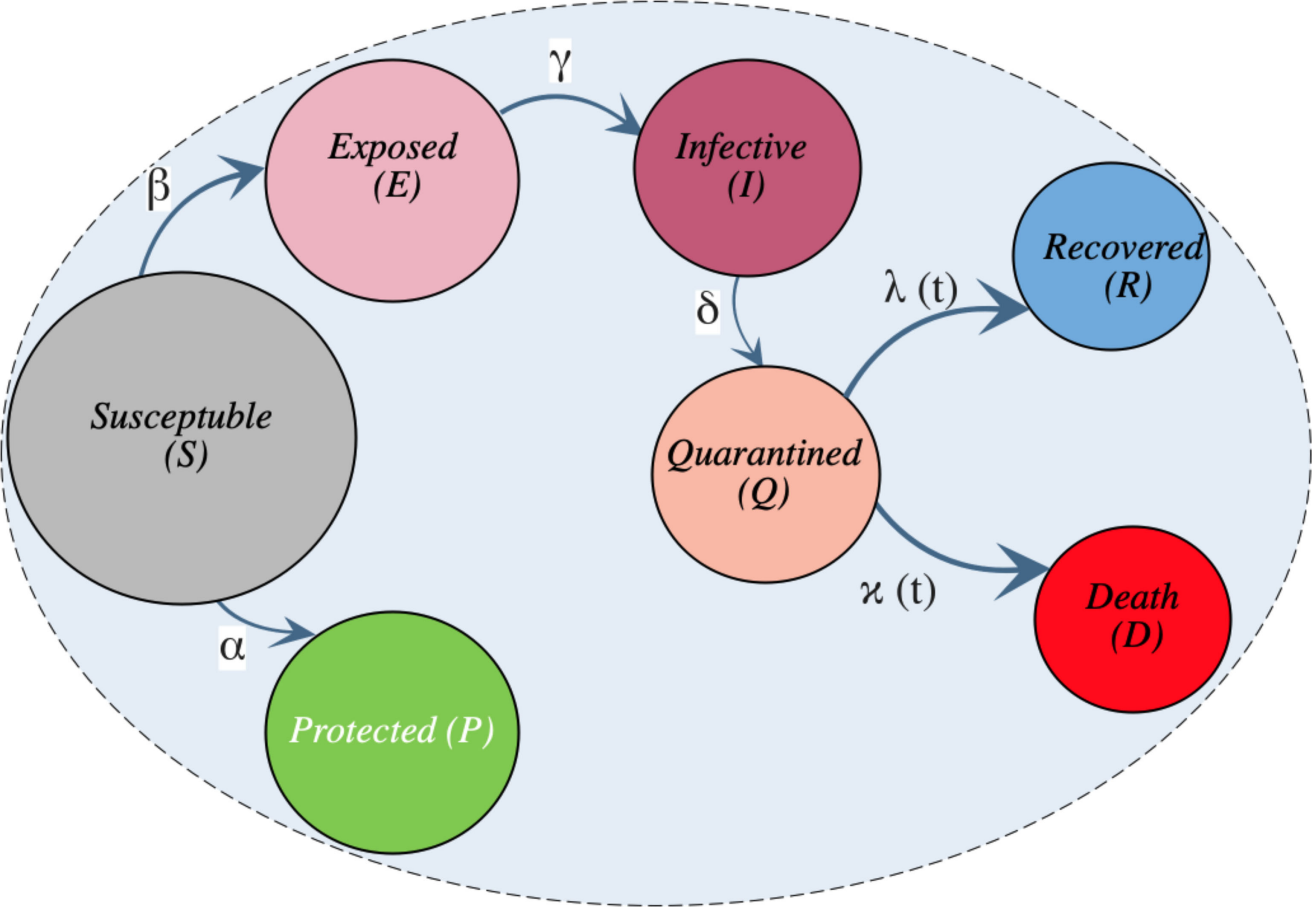
SEIQRDP compartment epidemic model showing the protection rate, $\alpha$, the infection rate, $\beta$, the inverse of the average latent time, $\gamma$, the rate at which infectious people enter in quarantine, $\delta$, the time dependent cure rate, $\lambda
                                                  (t)$, and the time dependent mortality rate $\kappa
                                                  (t)$.
• $\beta$: the infection rate.
• $\gamma$: the inverse of the average latent time.
• $\delta$: the rate at which infectious people enter in quarantine.
• $\lambda
                                            (t)$: a time-dependant coefficient used in the description of the cure rate. It is expressed as:
  \begin{equation*} \lambda (t) =\lambda
                                            _0 \left[1-e^{-\lambda _1t}\right], \tag{1}
                                            \end{equation*}
  where $\lambda
                                            _0$ and $\lambda
                                            _1$ are empirical coefficients.
• $\kappa
                                            (t)$: time-dependant coefficient used in the description of the mortality rate. It is expressed as:
  \begin{equation*} \kappa (t) =\kappa
                                            _0 \ e^{-\kappa _1 t}, \tag{2} \end{equation*}
  where $\kappa _0$ and $\kappa
                                            _1$ are empirical coefficients.

In the *SEIQRDP* epidemiological model, a population assumed constant is split into seven, disjoint, subpopulations based on their disease status. At time $t$: 1) the state $S(t)$ corresponds to the part of the population that is susceptible to COVID-19; 2) $E(t)$ represents the part of the population exposed to COVID-19; 3) $I(t)$ is the portion of the population designating individuals infectious with COVID-19; 4) $Q(t)$ is the fraction of the population representing quarantined individuals; 5) $R(t)$ is the part of population designating individuals that recovered from COVID-19, 6) $D(t)$ is the part of population designating individuals that dead from COVID-19, 7) $P(t)$ is the part of the population representing individuals that are insusceptible to COVID-19 (protected from COVID-19). Figure 1 illustrates the relations between all the fractions of the population. It shows the six rates defined as follows:

• $\alpha$: the protection rate.

It is worth to note that the time-dependent expressions of the cure rate, $\lambda
                            (t)$, and the mortality rate, $\kappa
                            (t)$, are assumed to be in the above forms based on the analysis of real data collected in some provinces in China, in January 2020, [23]. The plot and analysis of this data showed a gradual increase in the cure rate and a sharp decrease in the mortality rate. Furthermore, these assumptions are reasonable by nature as the function of death rate in such pandemics always converges to zero while the cure rate continues to increase toward a consistent level. The other parameters are assumed to be constant as they are not fluctuating over time. Generally, the above parameters depend on the application of preventive interventions and the effectiveness of the community's health systems.

The dynamic of each SEIQRDP's state is mathematically formulated using ordinary differential equations (ODE) as follows:

\begin{equation*} \left\lbrace
                                \begin{matrix}\frac{\mathrm{d}S(t)}{\mathrm{d}t} = -\alpha S(t)
                                -\beta \frac{S(t)I(t)}{N} \ \ \ \ \ \ \ \ \ \ \ \ \ \\ \\
                                \frac{\mathrm{d}E(t)}{\mathrm{d}t} = -\gamma E(t) +\beta
                                \frac{S(t)I(t)}{N} \ \ \ \ \ \ \ \ \ \ \ \ \ \\ \\
                                \frac{\mathrm{d}I(t)}{\mathrm{d}t} = \gamma E(t) -\delta I(t) \ \ \
                                \ \ \ \ \ \ \ \ \ \ \ \ \ \ \ \ \ \ \ \\ \\
                                \frac{\mathrm{d}Q(t)}{\mathrm{d}t} = \delta I(t) -\lambda (t)Q(t) -
                                \kappa (t) Q(t) \ \ \\ \\ \frac{\mathrm{d}R(t)}{\mathrm{d}t} =
                                \lambda (t) Q(t) \ \ \ \ \ \ \ \ \ \ \ \ \ \ \ \ \ \ \ \ \ \ \ \ \
                                \\ \\ \frac{\mathrm{d}D(t)}{\mathrm{d}t} = \kappa (t) Q(t) \ \ \ \ \
                                \ \ \ \ \ \ \ \ \ \ \ \ \ \ \ \ \ \ \ \\ \\
                                \frac{\mathrm{d}P(t)}{\mathrm{d}t} = \alpha S(t) \ \ \ \ \ \ \ \ \ \
                                \ \ \ \ \ \ \ \ \ \ \ \ \ \ \ \ \end{matrix}\right. \tag{3}
                                \end{equation*}

where $N$ represents the total population in the studied region expressed as $N =
                                S+E+I+Q+R+D+P$.

Comparing to the classical *SEIR* model, *SEIQRDP* is augmented by three new states, {*Q(t)*, *D(t)* & *P(t)*}. This new quarantined state *Q(t)* and the recovery state *R(t)* constitute, originally, the recovery state of the classical *SEIR* model [23].

**Fig. 2.**
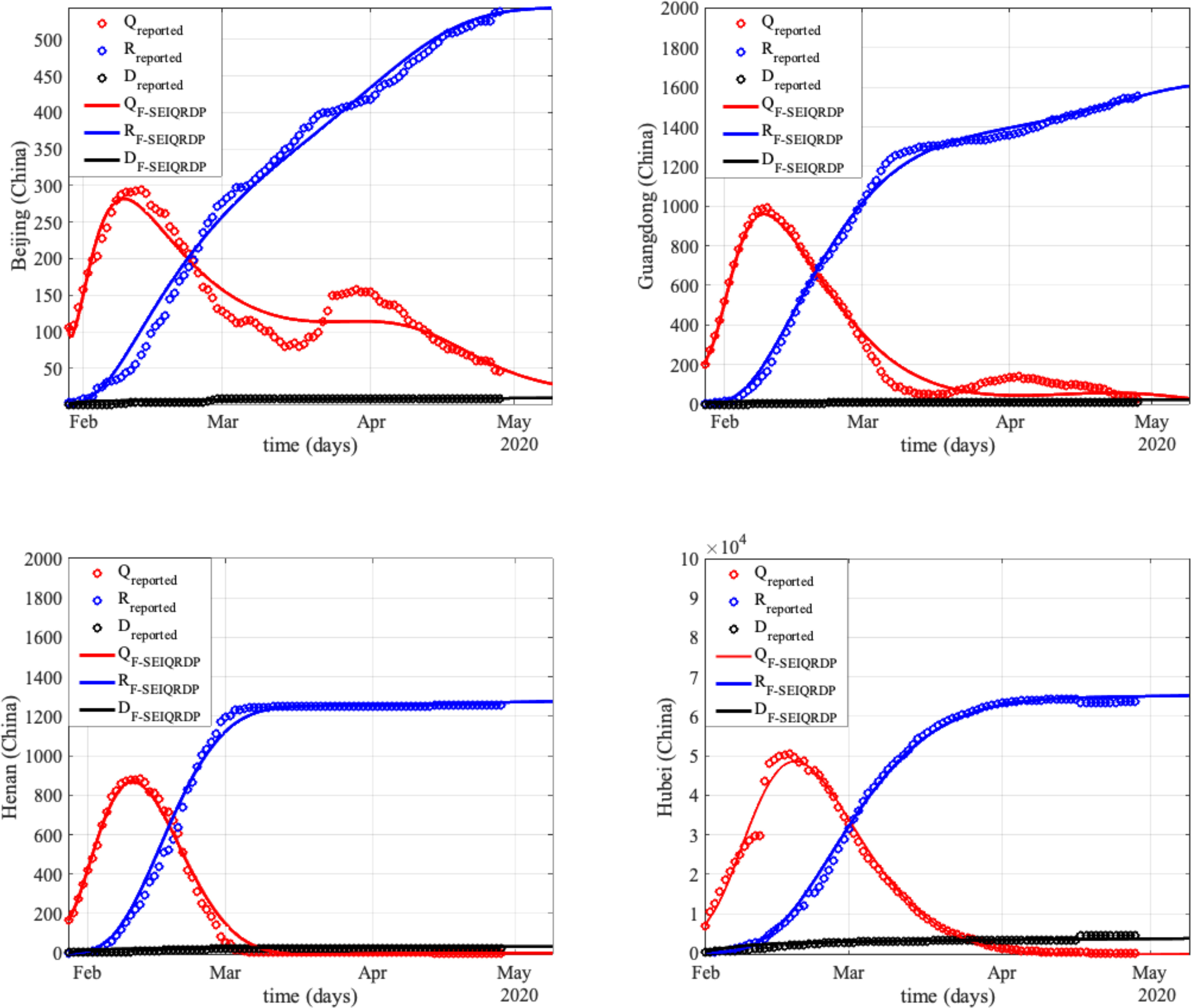
Predictions of the proposed fractional model using data from China.

**Fig. 3.**
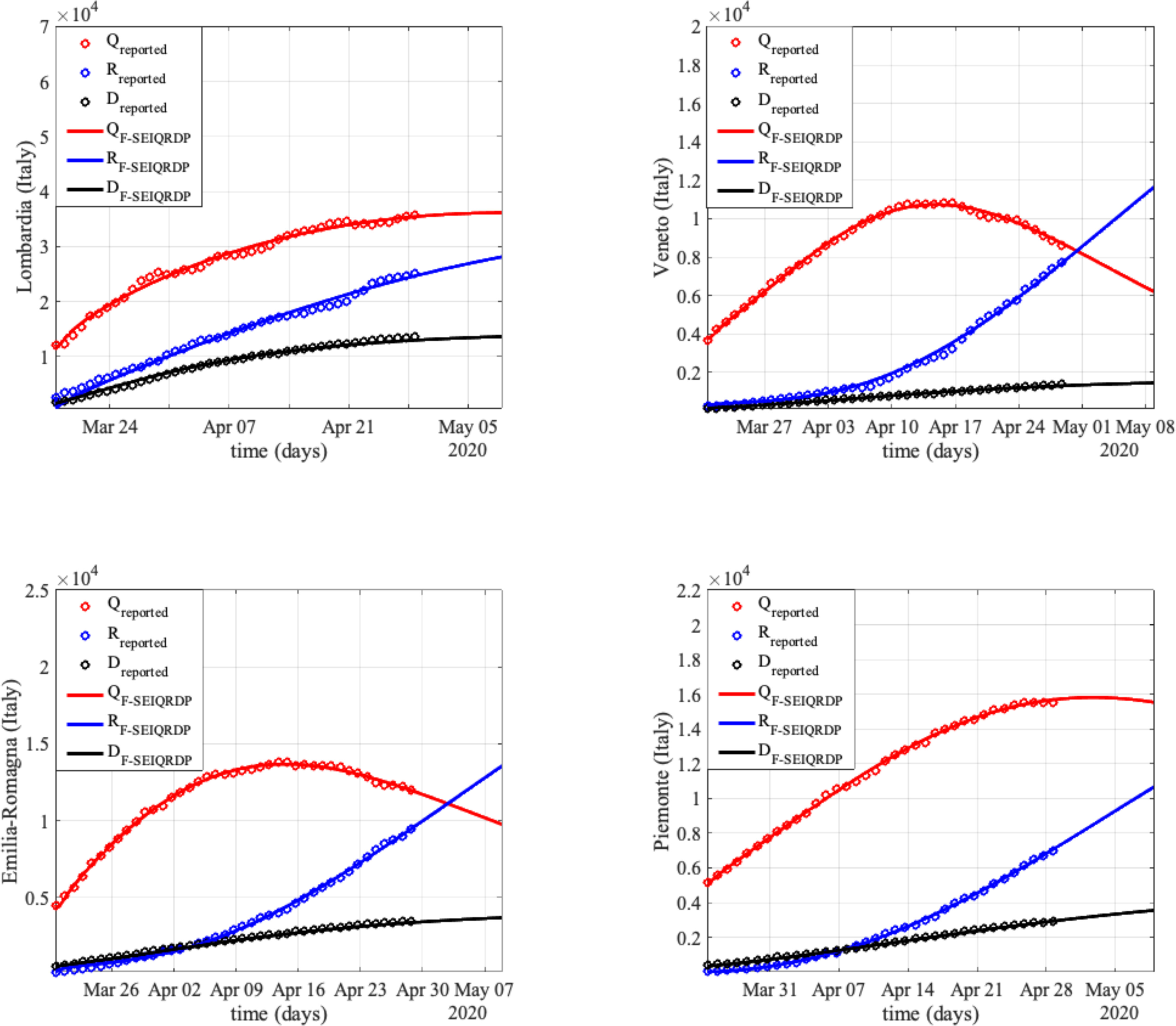
Predictions of the proposed fractional model using data from Italy.

**Fig. 4.**
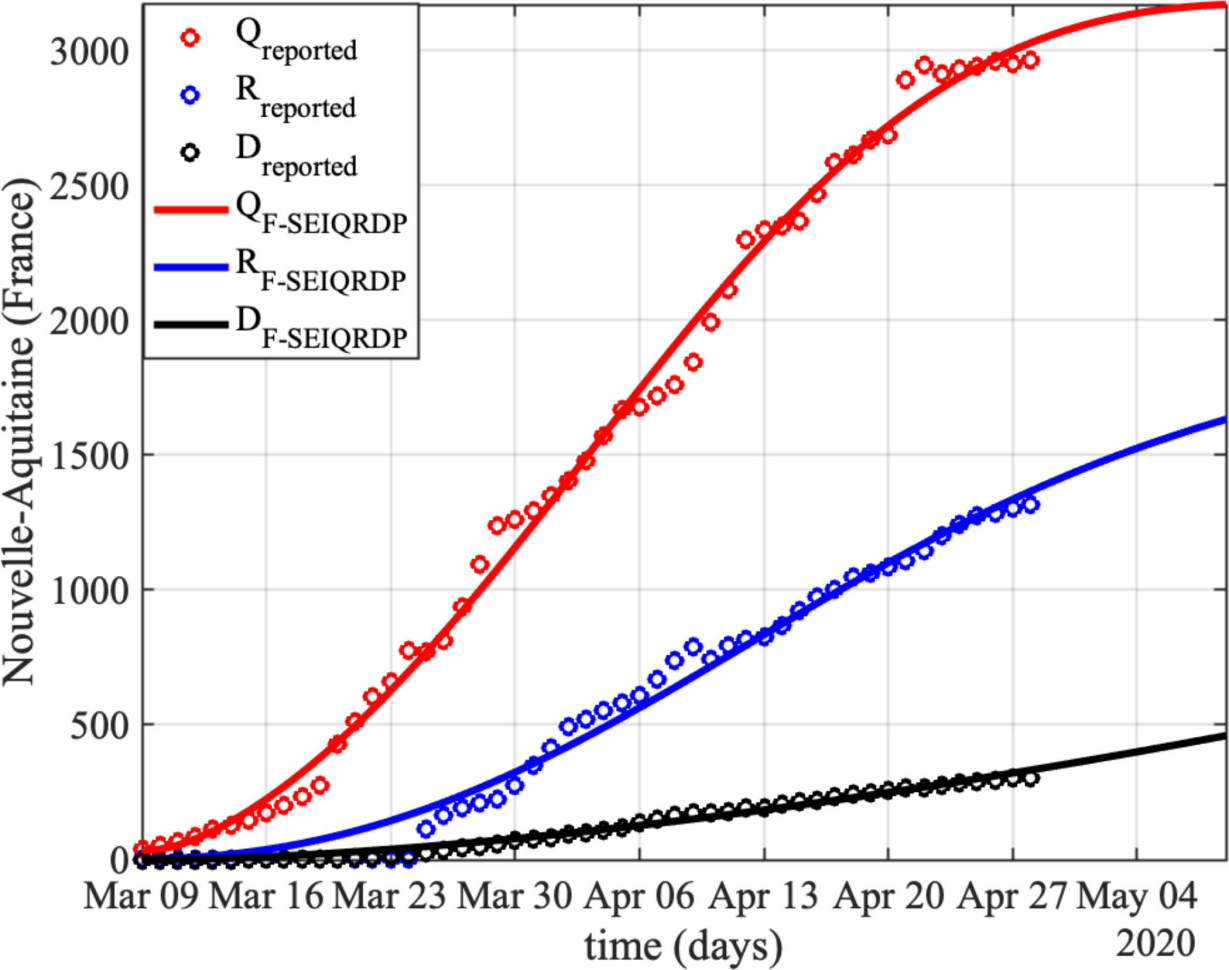
Predictions of the proposed fractional model using data from France.

#### 2) Fractional-order Derivative

In the past few decades, the theory of fractional calculus (FC) has gained significant research attention in several fields such as biology and epidemic modeling [24]–[26]. This is originated from the interdisciplinary nature of this field as well as the flexibility and effectiveness of FC in describing complex physical systems. For example, the characterization of bio-impedance, modeling of the viscoelasticity and biological cells, and representing the mechanical properties of the arterial system, as well as respiratory systems, have been investigated extensively through the exploring of FC [22], [27]–[30]. The concept of FC is not new dating from the pioneer conversation between *L’Hopital* and *Leibniz* in 1695 that yielded to the generalization of the conventional integer derivative to a non-integer order operator [31], as follows:

\begin{equation*} D^{q}_{t}= \left\lbrace
                                \begin{matrix}\frac{\mathrm{d^{q}} }{\mathrm{d} t^{\alpha }} &
                                \text{if} & q>0 \\ \\ 1,& \text{if} & q=0,\\ \\ \int
                                _{t}^{0}(df) ^{-q}& \text{if} & q< 0,\end{matrix}\right.
                                \tag{4} \end{equation*}

where ${q \in \mathbb
                                {R}}$ is the order of the operator known as the fractional-order, and ${df}$ is the derivative function.

There are several fractional-order derivative definitions. In this work, we introduce the three most frequently used ones in the sense of the *RiemannLiouville*, *Caputo* and *GrnwaldLetnikov* FD-based definitions [32]–[34]. The *GrnwaldLetnikov* scheme based on finite differences has been adopted in the numerical implementation of the proposed *F-SEIQRDP*.

For a function $g(t)$ that satisfies some smoothness conditions then:

• The *RiemannLiouville* definition is given as:

\begin{equation*}
                                _a^{RL}D_t^{q}g(t) =\frac{1}{\Gamma \left(n-q \right)
                                }\frac{\mathrm{d^n} }{\mathrm{d} t^n}\int _{0}^{t}\left(1-\tau
                                \right) ^{-q-1+n } g(\tau)d\tau . \tag{5} \end{equation*}

• The *Caputo* definition for FD is expressed as follows:

\begin{equation*}
                            _a^CD_t^{q}g(t) =\frac{1}{\Gamma \left(n-q \right) }\int
                            _{0}^{t}\left(1-\tau \right) ^{-q-1+n } \frac{\mathrm{d^n} }{\mathrm{d}
                            t^n}g\left(\tau \right) d\tau, \tag{6} \end{equation*}

where $\Gamma$ is the Euler gamma function and ($n-1< q<
                                n$).

• The GL definition is given as:

\begin{equation*}
                                _a^{GL}D_t^{q}g(t) =\lim _{h\to 0 }h^{-q}\sum
                                _{j=0}^{\left[\frac{t-a}{h}\right] }(-1)^j\binom{q}{j}g(t-jh),
                                \tag{7} \end{equation*}

where a is the terminal point and [.] means the integer part.

### B. Fractional-Order SEIQRDP Epidemiological Model (F-SEIQRDP)

Similar to the *SEIQRDP*, [23], the *F-SEIQRDP* epidemiological model considers that the total population ($N$) is divided into seven sub-populations i.e., $\lbrace
                            S(t),E(t),I(t),Q(t),R(t),D(t),P(t)\rbrace$.

As the fractional-order derivative takes into account the history of the state, we believe that this operator is more suitable to describe the dynamics of the epidemic COVID-19. Using the definition of the fractional-order derivative operator, we consider that each state follows a fractional-order behavior.

Considering the nonlinear FODEs in this matrix form:

\begin{equation*} D_t^q X(t)=A X(t)+L(X),
                            \tag{8} \end{equation*}

where,

\begin{equation*}
                            D^q=[D^{q_S},D^{q_E},D^{q_I},D^{q_Q},D^{q_R},D^{q_D},D^{q_P}]^T
                            \end{equation*}

is the fractional-order derivative operator for all the states and

\begin{equation*}
                            X=[S,E,I,Q,R,D,P]^T \end{equation*}

represents the state vector.

\begin{equation*} A= \begin{bmatrix}-\alpha ^{q_S}
                            & 0 & 0 & 0 & 0 & 0& 0\\ 0& -\gamma ^{q_E}
                            & 0 &0 & 0 & 0&0 \\ 0& \gamma ^{q_I} &
                            -\delta ^{q_I}& 0 & 0& 0& 0\\ 0& 0& \delta
                            ^{q_Q}& -\kappa ^{q_Q}(t)-\lambda ^{q_Q}(t) & 0& 0 & 0\\
                            0& 0 & 0&\lambda ^{q_R}(t) & 0 & 0 &0 \\ 0&
                            0 & 0 & \kappa ^{q_D}(t) & 0 & 0 &0 \\ \alpha
                            ^{q_P}& 0& 0 & 0 & 0&0 &0 \end{bmatrix}
                            \end{equation*}

represent the parameters.

$L(X)$ depicts the nonlinear term that is function of the susceptible and

\begin{equation*} L=S(t) I(t)
                            \begin{bmatrix}-\frac{\beta ^{q_S}}{N}\\ \\ -\frac{\beta ^{q_E}}{N}\\ \\
                            0\\ 0\\ 0\\ 0\\ 0 \end{bmatrix} \end{equation*}

### C. Dataset

The updated epidemic data of different countries around the world is collected from authoritative and known sources as follows:

• **France**: The data is gathered from three main sources: “Agence Regionale de Sante,” “Sant Publique France” and “Geodes”. This data is publicly available.^1^^1^https://www.github.com/cedricguadalupe/FRANCE-COVID-19.
• **Italy**: This data is provided by the Italian government and it is publicly available.^2^^2^https://github.com/pcm-dpc/COVID-19.
• **Other countries**: The data is gathered from different official sources: World Health Organization (WHO), Center of Disease Control and Prevention (CDC), the COVID Tracking Project (testing and hospitalizations), etc. The data repository is operated by the Johns Hopkins University Center for Systems Science and Engineering (JHU CSSE) and Supported by ESRI Living Atlas Team and the Johns Hopkins University Applied Physics Lab (JHU APL). The repository is publicly available.^3^^3^https://github.com/CSSEGISandData/COVID-19.

### D. Data Fitting Algorithm and Numerical Simulations

The parameters of the proposed fractional-order model were estimated by a non-linear least square minimization routine, making use of the well-known $\mathrm{MATLAB-R2019b}$, function *lsqnonlin*. This function is based on the trust-region reflective method [35]. The steps used to obtain the optimal estimates are outlined in Algorithm 1.

Algorithm 1: **Parameter Estimation of Epidemic Data.**
Input:
$t$: Time in days
$R$: Recovered cases
$I$: Confirmed cases
$D$: Dead cases
$guess$: The initial guess of the parameters
$funmodel$: The model to be fitted
Output:
$param$: Fitted parameter of the $funmodel$
*- Set the initial conditions*
E = I; $\triangleright$ Unknown but unlikely to be zero.
Q = I-R-D;
input = [E; I; Q; R; D]
*- Run the fitting optimization*
param = lsqcurvefit (t, *funmodel*, *guess*, *input*)

The fitting performances are evaluated using the followings metrics:

\begin{equation*} RMSE
                            = \sqrt{\frac{1}{l}\Sigma _{i=1}^{l}{\left({y(i)
                            -\hat{y}(i)}\right)^2}}, \tag{9} \end{equation*}

and

\begin{equation*} ReMSE
                            = \frac{ \frac{1}{l} \Sigma _{i=1}^{l}{| {y(i) -\hat{y}(i)}|}}{\max(y)},
                            \tag{10} \end{equation*}

where $y$ and $\hat{y}$ are the real and fitted data, respectively. $l$ is the length of the data. The MATLAB code can be downloaded from URL https://github.com/EMANG-KAUST/COVID-19.

## III. Results

The fitting performance of predicting the dynamics of $Q(T)$, $R(t)$, and $D(t)$ populations using *SEIQRDP* and the proposed *F-SEIQRDP* are presented in Table I. It is worth to note that *SEIQRDP* epidemiological model can be considered as a particular case of the *F-SEIQRDP*, where all the fractional differentiation orders are equal to 1. Accordingly, the proposed fractional-order paradigm can be observed as a generalized framework of SEIQRDP epidemiological modeling. From the reported results, it is clear that for all the studied regions, the $\mathrm{RMSE}$ as well as the $\mathrm{ReRMSE}$ based on *F-SEIQRDP* model are less than the ones reported using *SEIQRDP*. These results show the usefulness of the fractional-order derivative operator in fitting real data of the pandemic. Besides, it demonstrates the potential of the fractional-order framework in estimating the size and the critical milestones of the spread of the epidemic-COVID-19. The appropriateness of the fractional-order paradigm can be reflected from the fact that: FD operator is not local and depends on the strength of the memory that is controlled by the fractional differentiation order. On the other hand, the epidemiological dynamical process is involving the memory effect within the sub-diffusion process of confirmed and recovered cases growth. The parameter estimates for all the studied populations in different regions are reported in Table II. In this study, we choose different regions that present different circumstances in terms of population, the number of infected cases, and the lock-down schemes. Figures 2, 3 and 4 show examples of the predicted dynamic of the quarantined, recovered and death sub-populations in {Beijing, Guangdong, Henan, and Hubei} regions in China, {Lombardia, Veneto, Emilia-Romagna, and Piemonte} regions in Italy, and {Nouvelle Aquitaine} region in France, respectively. It is clear in all the figures that we present the trend of the epidemiological dynamic till May $8^{th}$, which is the future concerning the date of simulation April, $29^{th}$. This shows the potential of the model in predicting the trend of the pandemic dynamic in the future.

**TABLE I:** Estimation Error Comparison Between the SEIQRDP Model and the Proposed F-SEIQRDP Model for Different Countries

**Model**	**Estimation Error**	**China**				**Italy**				**France**
		Beijing	Guangdong	Henan	Hubei	Lombardia	Veneto	Emilia- Romagna	Piemonte	Nouvelle- Aquitaine
**F-SEIQRDP**	$\mathrm{ReMSE}$	0.0625	0.0420	0.0215	0.0191	0.0145	0.0071	0.0063	0.0048	0.0159
	$\mathrm{RMSE}$	22.17	53.26	30.56	1677.89	648.37	97.21	108.912	97.96	63.94
**SEIQRDP**	$\mathrm{ReMSE}$	0.1388	0.1053	0.0315	0.0287	0.0169	0.0093	0.0065	0.0047	0.0231
	$\mathrm{RMSE}$	45.84	132.02	45.51	2063.95	732.878	119.08	114.67	97.00	85.44

**TABLE II:** Epidemic Spread Parameters Estimation Using the Proposed F-SEIQRDP Model for Different Countries

**Country**	**China**				**Italy**				**France**
**City**	*Beijing*	*Guangdong*	*Henan*	*Hubei*	*Lombardia*	*Veneto*	*Emilia- Romagna*	*Piemonte*	*Nouvelle Aquitaine*
Population rate (million)	21.54	113.46	94	58.50	10.04	4.90	4.45	4.37	5.98
Protection rate $ (\alpha)$	0.03820	0.03423	0.57635	0.23436	6.7E-5	0.05720	0.02336	0.02087	0.01251
Infection rate $ (\beta)$	0.00261	0.00105	0.00626	0.01940	0.8820	0.83078	0.72296	0.86569	0.50255
Latent time $ (\gamma ^{-1})$	3.44862	2.92057	4.38832	4.88390	7.1748	7.34966	4.27373	8.34334	2.73742
Quarantine time $ (\delta ^{-1})$	4.06448	4.76581	6.20915	6.59052	1.9342	1.84847	2.19720	2.15438	2.59576
**$\lambda _0$**	0.06655	0.12695	0.70887	0.33212	0.0393	0.32064	0.03188	0.09577	0.00390
**$\lambda _1$**	0.05342	0.02932	0.00585	0.00623	0.0036	0.00361	0.07352	0.00582	0.00839
**$\kappa _0$**	0.02674	0.05612	0.05950	0.05010	0.0342	0.00640	0.01320	0.01051	0.22037
**$\kappa _1$**	0.68415	0.85788	0.67755	0.31469	0.0634	0.02783	0.03674	0.02526	0.37194
**$q_S$**	2.06	2.31	0.40	0.89	0.89	1.09	1.00	0.99	0.98
**$q_E$**	1.26	1.37	1.20	1.36	0.84	0.73	0.79	0.76	1.40
**$q_I$**	0.88	0.93	1.28	1.17	0.84	0.72	0.87	0.70	0.83
**$q_Q$**	0.87	1.04	1.03	1.06	0.86	0.94	0.88	0.94	0.92
**$q_R$**	0.87	0.99	1.04	1.05	0.23	1.14	1.30	0.71	0.24
**$q_D$**	1.20	1.20	1.20	1.20	1.20	1.20	1.0	1.0	2.59
**$q_P$**	1.00	1.00	1.00	1.00	1.00	1.00	.00	1.00	1.00

## IV. Conclusion

In epidemiology, mathematical modeling is concerned with representing the spread of infectious illness and its effect on the community. Consequently, analytical models are deemed essential in every stage of pandemic development. The simulation of the epidemic dynamics helps to understand and monitor the spread of the infection. Furthermore, models are beneficial in estimating the size of the pandemics, so they help the specialized health actors to make the right decisions and lessen the damages. In this paper, we propose a novel general fractional-order model for the evolution of the COVID-19 pandemic. The validation results show the accurate model fitting using real COVID-19 data from various countries’ regions. The fractional-order derivatives provide new parameters for the control of the epidemic, offering extra flexibility. However, the model has some limitations that we can list as follows.

• We observe that the estimation is less accurate for countries with limited data (reported cases in less than 30 days) because the trend of the pandemic does not appear yet.
• From country to country, the initial guess of parameters should be chosen carefully to guarantee the best possible fitting of the employed optimization solver.
• The time-dependent parameters (the mortality rate $\kappa
                                    (t)$ and the cure rate $\lambda
                                    (t)$) need to be reconsidered carefully because the countries do not have similar medical facilities and expertise and perform different numbers of tests per day. Besides, some countries have adopted some precautions strategies earlier than others such as quarantine, lock-down...etc.

Interestingly, in the future, the above limitations should be studied and taken into consideration. Furthermore, conducting intensive local/global sensitivity analysis and computational simulations of the proposed model is necessary. It will be handy for future model integration within control strategies of the pandemic, interventions, especially during vaccination programs.
